# Molecular Typing of *Mycobacterium bovis* from Cattle Reared in Midwest Brazil

**DOI:** 10.1371/journal.pone.0162459

**Published:** 2016-09-15

**Authors:** Ricardo César Tavares Carvalho, Sidra Ezidio Gonçalves Vasconcellos, Marina de Azevedo Issa, Paulo Martins Soares Filho, Pedro Moacyr Pinto Coelho Mota, Flábio Ribeiro de Araújo, Ana Carolina da Silva Carvalho, Harrison Magdinier Gomes, Philip Noel Suffys, Eduardo Eustáquio de Souza Figueiredo, Vânia Margaret Flosi Paschoalin

**Affiliations:** 1 Instituto de Química, Universidade Federal do Rio de Janeiro (UFRJ), Rio de Janeiro/RJ, Brasil; 2 Laboratório de Biologia Molecular Aplicado a Micobactérias, Instituto Oswaldo Cruz (IOC), Fundação Oswaldo Cruz (FIOCRUZ), Rio de Janeiro/RJ, Brasil; 3 Mycobacteriology Unit, Tropical Institute of Medicine, Antwerp, Belgium; 4 Laboratório Nacional Agropecuário (LANAGRO), Ministério da Agricultura, Pecuária e Abastecimento (MAPA), Pedro Leopoldo/MG, Brasil; 5 Empresa Brasileira de Pesquisa Agropecuária (EMBRAPA Gado de Corte), Campo Grande/MS, Brasil; 6 Universidade Federal do Rio de Janeiro (UFRJ)—Campus Macaé, Macaé/RJ, Brasil; 7 Faculdade de Nutrição, Universidade Federal de Mato Grosso (UFMT), Cuiabá/MT, Brasil; University of Minnesota, UNITED STATES

## Abstract

*Mycobacterium bovis* is the causative agent of bovine tuberculosis (BTB), the pathogen responsible for serious economic impact on the livestock sector. In order to obtain data on isolated *M*. *bovis* strains and assist in the control and eradication program for BTB, a cross sectional descriptive molecular epidemiology study in the Brazilian Midwest was conducted. Through spoligotyping and 24-*loci* MIRU-VNTR methods, 37 clinical isolates of *M*. *bovis* circulating in the region were analyzed, 10 isolated from the state of Mato Grosso, 12 from the state of Mato Grosso do Sul and 15 from the state of Goiás. The spoligotyping analysis identified 10 distinct *M*. *bovis* profiles (SB0121 n = 14, SB0295 n = 6, SB0140 n = 6, SB0881 n = 3, SB1144 n = 2, SB1145 n = 2, SB0134 n = 1, SB1050 n = 1, SB1055 n = 1, SB1136 n = 1) grouped in six clusters and four orphan patterns. The MIRU-VNTR 24-*loci* grouped the same isolates in six clusters and 22 unique orphan patterns, showing higher discriminatory power than spoligotyping. When associating the results of both techniques, the isolates were grouped in five clusters and 24 unique *M*. *bovis* profiles. Among the 24-*loci* MIRU-VNTR evaluated, two, ETR-A and QUB 11b *loci*, showed high discriminatory ability (h = ≥ 0.50), while MIRU 16, MIRU 27, ETR-B, ETR-C, Mtub21 and QUB 26 *loci* showed moderate ability (h = 0.33 or h = 0.49) and were the most effective in evaluating the genotypic similarities among the clinical *M*. *bovis* isolate samples. Herein, the 29 patterns found amongst the 37 isolates of *M*. *bovis* circulating in the Brazilian Midwest can be due to the animal movement between regions, municipalities and farms, thus causing the spread of various *M*. *bovis* strains in herds from Midwest Brazil.

## Introduction

*Mycobacterium bovis* is a bacteria belonging to the *Mycobacterium tuberculosis* complex (MTC), which, in addition to causing tuberculosis in cattle and buffaloes (BTB), can cause disease in several species of mammals, including humans, thus being considered a zoonosis [1,2].

BTB is a worldwide-distributed disease with striking prevalence in developing countries. This disease has socio-economic impacts by reducing livestock productivity due to early disposal of high zootechnical value animals, reduction in weight gain of affected animals and loss in the export of products from the cattle industry, mainly meat [3,4].

Infection by *M*. *bovis* in humans is typically caused by the consumption of animal food products contaminated by the bovine bacillus, usually unpasteurized milk and milk derivatives [5], leading to the development of tuberculosis in its extrapulmonary form [6]. Another route for *M*. *bovis* infection in humans is through airborne transmission [7,8]. These infections are clinically and pathologically indistinguishable from tuberculosis (TB) caused by *M*. *tuberculosis* [9,6]. It is suspected that infections caused by *M*. *bovis* are responsible for more than 4000 cases among the 100,000 cases of human tuberculosis described annually in Brazil [10,11]. However, according to the World Organization for Animal Health (OIE), the number of human TB cases caused by *M*. *bovis* in Brazil cannot be estimated [12], since bacteriological culture followed by biochemical identification tests to diagnose whether the infective agent was *M*. *bovis* or *M*. *tuberculosis* are not performed in most tuberculosis cases [13].

Cattle raising is very important for the Brazilian economy. Currently, the cattle herd in the country is over 212 million heads, and the Midwestern region, formed by the states of Mato Grosso, Mato Grosso do Sul and Goiás, is the main cattle-producing region [14] and the largest beef exporting region in the country [15]. Although livestock sanitary risks could impact the agribusiness on Brazilian economy, there is still a lack of updated data on the distribution and prevalence of BTB in the country and in the different producing regions. The latest official national prevalence data of the disease was in 2004, reporting a rate of 1.3% [8]. On the other hand, the estimated prevalence of the disease in the Midwest was of 0.37%, as described by Roxo, in 2004 [16]. In a recent study, the estimated prevalence of BTB for the state of Mato Grosso, which is part of the Midwest region, was estimated at 0.007% [17]. It is believed that, currently, the prevalence of BTB in the whole Midwestern region may be lower than that described in 2004 [16].

In order to reduce the prevalence and incidence of new BTB outbreaks in herds, to certify properties as free or monitored for the disease, and to offer consumers lower health risk products, Brazil, the Ministry of Agriculture Livestock and Supply (MAP) launched the National Program for Control and Eradication of Bovine Brucellosis and Tuberculosis (PNCEBT) [8] in 2001, which was regulated in 2004. This animal health program recommends performing the intradermal tuberculin test, followed by the slaughter of positive cattle, surveillance in slaughterhouses, tracing the origin of the outbreak and sanitation, as established by the International Organization for Animal Health [18].

The molecular identification of strains involved in BTB infection may contribute to an increased efficiency of disease control programs, since the identification of *M*. *bovis* genotypes prevalent in a particular area, allows to track and control the occurrence of multiple foci of disease [19,20], especially in areas with low prevalence of the disease, as is the case of the Brazilian Midwestern region.

Spacer oligotyping (spoligotyping) and variable number tandem repeat (VNTR) are amply used techniques in human tuberculosis epidemiological studies, as well as molecular typing of MTC species, which includes *M*. *bovis* [21]. When combined, spoligotyping and VNTR are able to distinguish the bacteria lineages more effectively [22,23,24], with a good cost/benefit relationship, due to speed, reproducibility and reliability of the performed genotyping [25,26,27,28].

The MIRU-VNTR is based on the size analysis of amplified fragments from multiple *loci*, determining the number of repetitions of each *locus* [29,30,31,32]. The analysis of the amplified fragment can be done manually by agarose gel electrophoresis [33] or automatically by capillary electrophoresis [34]. Each technique has its advantages and disadvantages that must be considered when choosing which to implement in the laboratory. Spoligotyping in combination with MIRU-VNTR analysis seems to be the best choice, since both have the advantage of being PCR-based, and, when combined, discriminatory power is improved [19].

In this context, a cross sectional study of molecular epidemiology was conducted for the characterization of *M*. *bovis* isolates circulating in the Brazilian Midwest and the comparison with *M*. *bovis* strains from other regions of Brazil and the world was performed.

## Materials and Methods

### Bacterial isolates and DNA extraction

The present study was based on a convenience sampling of BTB diagnosed between 2010 to 2013, at the National Agricultural Laboratory (LANAGRO/MAPA/BRASIL). A total of 37 *M*. *bovis* isolates were obtained from clinical samples taken from suspected BTB lesions from 37 animals that scored positive in the intradermal tuberculin test in the Brazilian Midwest region (Mato Grosso, Mato Grosso do Sul and Goiás). These isolates were previously identified by biochemical [26] and molecular tests [4]. DNA templates were extracted by the thermal lysis method [35] and purified using the commercial kit ChargeSwitch^®^ PCR Clean-up kit (Invitrogen, CA, USA). DNA templates from *M*. *bovis* BCG and *M*. *tuberculosis* H37Rv were used as positive controls in the spoligotyping and MIRU-VNTR assays.

### Spoligotyping

The spoligotyping method was conducted as described by Kamerbeek et al. (1997) [28]. Hybridisation of the PCR product to the spoligo-membrane was performed according to the manufacturer’s instructions (Ocimum Biosolutions, Telangana, IN). Bound fragments were detected by chemiluminescence after incubation with peroxidase-labelled streptavidin (1:4000). Only patterns with 100% similarity were considered as clusters. Those strains clustered by spoligotyping were analyzed by MIRU-VNTR to confirm their clonal relationships. *M*. *bovis* profiles were compared to those available at the Mbovis.org website (http://www.mbovis.org/) [36] and SITVIT-WEB (http://www.pasteur-guadeloupe.fr:8081/SITVIT_ONLINE/) databases.

### MIRU-VNTR typing

*M*. *bovis* strain typing was carried out by MIRU-VNTR automated in-house technique, according to De-Beer et al. (2012) [37] with modifications. The detection of 24-*loci* MIRU-VNTR labeled with fluorophores (6FAM^™^/green, VIC^®^/blue and NED^™^/yellow) was performed, as recommended by Supply et al. (2006) [32]. For each sample, eight PCRs were carried out, using three primer pairs (*triplex*-PCR) each for the simultaneous amplification of three distinct *loci* [32].

*Tríplex-*PCR was performed using 0.4 μl of each primer (Applied Biosystem, CA, USA), at the concentrations described by Supply et al. (2006) [32], 1X KAPA2G Fast HotStar ReadMix PCR Kit^®^ (Kapabiosystems, MA, USA), 1.87 μl of DMSO [p.a.] and 2 μl of purified DNA (about 20 ng) in a final volume of 20 μl. PCR assay conditions were 3 min at 95°C, followed by 30 cycles for 15 sec at 95°C, 15 sec at 59°C, 30 sec at 72°C and a final extension step at 72°C for 10 min.

PCR products (1 μl) were prepared for automated fragment reading on an optical plate—MicroAmp^®^ Optical 96-well Reaction (Applied Biosystem, CA, USA) by adding 0.4 μl of the molecular marker GeneScan™ 1200 LIZ^®^ Size Standard (Applied Biosystem), 8.6 μl Hidi formamide (Applied Biosystems) in a final volume of 10 μl. All mixtures were denatured at 95°C for 2 min and immediately cooled on ice. The fragment size of the amplicons was analyzed on a ABI 3130*xl* DNA sequence analyzer (Applied Biosystems) and the number of copies of each *locus* was determined by automated assignment using the GeneMapper^®^ 4.0 software (Applied Biosystems). In case of doubtful results, the length of the repeats was double checked by size fragment estimation as compared to a DNA ladder (50 and 100 bp). Aplicons from *M*. *bovis* BCG and H37Rv strains were compared with the reference table described by Supply et al. (2000) [31].

The sample profiles were compared to those available at the database MIRU-VNTR plus (http://www.miru-vntrplus.org/MIRU/index.faces) and analyzed by *BioNumerics* software 6.6 (Applied Maths, Sint-Martens-Latem, BE).

### Allelic and genotypic diversity calculations

The Hunter-Gaston discriminatory index (HGDI) [38] was used to calculate the allelic diversity within each MIRU-VNTR *locus* and the genotypic diversities (discriminatory power) of the spoligotyping assays, 24-MIRU-VNTR and the combination of both methodologies.

### Clustering analysis

The number and fragment length of the genotype clusters were introduced as numerical data into an Excel spreadsheet template and different criteria for definition of the clusters were used, such as the analysis of individual spoligotyping or combination of results from spoligotyping and MIRU-VNTR. Data were analyzed by the *BioNumerics* software 6.6 (Applied Maths, East Flanders, BE) in order to construct the similarity matrices and the dendrogram (unweighted pair-grouping method analysis algorithm—UPGMA).

## Results and Discussion

**Fig 1:**
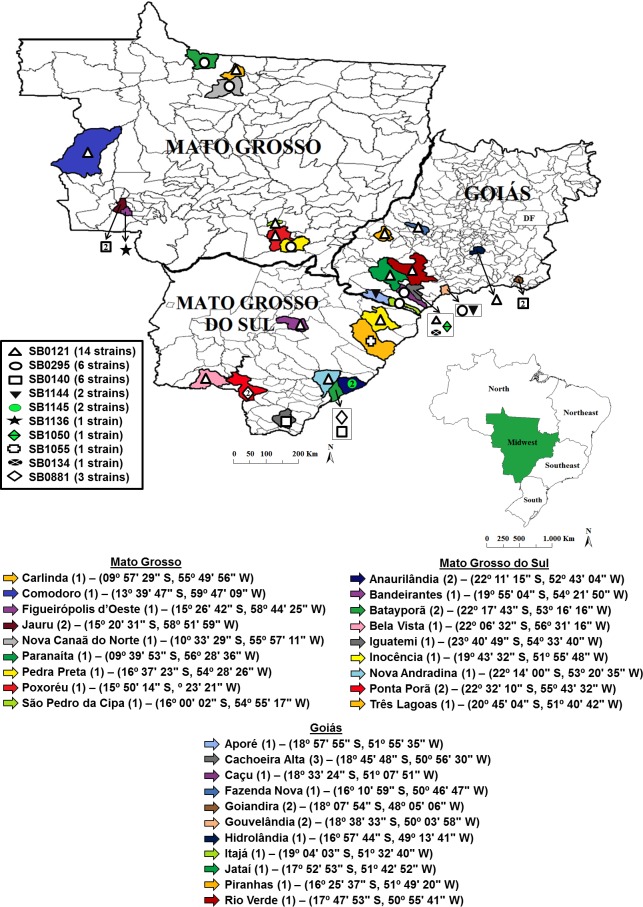
Geographic origin of each *M*. *bovis* spoligotype found in strains isolated at municipalities of Midwest Brazil.

After the spoligotyping, the 37 *M*. *bovis* isolates were classified as (Table 1) SB0121 (n = 14; 37.8%), SB0295 (n = 6, 16.2%), SB0140 (n = 6), SB0881 (n = 3, 8.1%), SB1144 (n = 2, 5.4%) and SB1145 (n = 2). In addition, four strains (10.8%), SB0134, SB1050, SB1055 and SB1136, showed orphan patterns. The geographic distribution of the spoligotypes is presented in Fig 1.

**Table 1:** Molecular characterization of the 37 *M*. *bovis* isolates by spoligotyping method.

Sample	Spoligotype	Spoligotype pattern
44	1100000101111110111101111000011111111100000	SB1145
45	1100000101111110111101111000011111111100000	SB1145
49	1101111101111110111101111000000111111100000	SB0881
52	1101111101111110111101111000000111111100000	SB0881
10	1101111101111110111101111000000111111100000	SB0881
35	1101111101111110111101111111111111111100000	SB0121
36	1101111101111110111101111111111111111100000	SB0121
11	1101111101111110111101111111111111111100000	SB0121
22	1101111101111110111101111111111111111100000	SB0121
23	1101111101111110111101111111111111111100000	SB0121
30	1101111101111110111101111111111111111100000	SB0121
37	1101111101111110111101111111111111111100000	SB0121
33	1101111101111110111101111111111111111100000	SB0121
39	1101111101111110111101111111111111111100000	SB0121
48	1101111101111110111101111111111111111100000	SB0121
17	1101111101111110111101111111111111111100000	SB0121
5	1101111101111110111101111111111111111100000	SB0121
4	1101111101111110111101111111111111111100000	SB0121
38	1101111101111110111101111111111111111100000	SB0121
15	1101111101111110111101111111111111110100000	SB0295
16	1101111101111110111101111111111111110100000	SB0295
13	1101111101111110111101111111111111110100000	SB0295
12	1101111101111110111101111111111111110100000	SB0295
25	1101111101111110111101111111111111110100000	SB0295
18	1101111101111110111101111111111111110100000	SB0295
1	1101111101111110111101111111100000001100000	SB1144
21	1101111101111110111101111111100000001100000	SB1144
19	1101101000001110111111111111111111111100000	SB0140
20	1101101000001110111111111111111111111100000	SB0140
27	1101101000001110111111111111111111111100000	SB0140
28	1101101000001110111111111111111111111100000	SB0140
46	1101101000001110111111111111111111111100000	SB0140
29	1101101000001110111111111111111111111100000	SB0140
14	1100011101111110111111111111111111111100000	SB0134
24	0000000000011110111111111111111111111100000	SB1136
3	1101111101111110111101111111100000111100000	SB1050
9	1100011101111110111111111111111111110100000	SB1055
*M*. *bovis BCG*	1101111101111110111111111111111111111100000	Reference strains
*M*. *tuberculosis* H37Rv	1111111111111111111001111111111100001111111	Reference strains

The predominant spoligotype SB0121 was widespread in the three states of the Brazilian Midwest, and has also been described as the most prevalent in other Brazilian regions, including in the states of Rio Grande do Sul (92.9%), in the Southern region of the country [19], São Paulo (32.7%) [39] and Minas Gerais (16.4%) [40], both in the Southeastern region, in the state of Bahia (36%), in the Northeast, [3] and in the state of Mato Grosso do Sul (30.7%), in the Midwest [20]. Outside Brazil, SB0121 has been described in the Netherlands [41], France [41,42], Italy [43], Belgium [41], Portugal [44], Spain [45], Algeria [46], South Africa [47], Mexico [48,49] and Venezuela [49]. Interestingly, the SB0121 spoligotype has not yet described in Argentina, a country that borders Brazil and where animal movement between the countries frequently occurs [49].

The second most frequent spoligotype, SB0295, found in Mato Grosso and Goiás has been described in the states of São Paulo (35%) [39] and, Bahia (14%) [3], consistent with the national prevalence of 24% [49]. The SB0295 spoligotype has also been described in Spain [50], Portugal [44], France [42] and Mexico [51].

Spoligotypes SB0121 and SB0295 differ by one spacer only in the DR (direct repeat) region (Table 1) and were presently responsible for 54% genotypes of the strains isolated from Midwestern Brazil. The small discrepancy in these spoligotypes may be associated with strains that have undergone genetic mutation, which may cause difficulties in BTB diagnostics through the conventional tuberculin test, adopted throughout the country for BTB control in cattle herds [19,52,53]. Infections caused by strains classified as SB0121 and SB0295 spoligotypes occurred in municipalities very near to each other and suggests a selection of these lineages in these geographic locations (Fig 1).

Although spoligotype SB0140 was observed at a lower frequency (16.2%), it occurred in the three investigated states and was found with similar a frequency in São Paulo [54]. It has also been described throughout the four continents, in several countries, including Mexico [48,49,51], Argentina [49,55], Paraguay [55], Uruguay [55] Chile [49], France [41], Italy [43], Ireland [56,57], United Kingdom [58,59], South Africa [47] and Australia [56].

The SB0881 spoligotype was identified only in Mato Grosso do Sul (Fig 1) and is the third most prevalent in Brazil [49], having previously been reported in the country [20,39,40], and having also been shown to occur in Spain [45] and in France [41].

The SB1144 and SB1145 spoligotypes were identified in only two isolates each, the former in Goiás and the latter in Mato Grosso do Sul (Fig 1). These spolygotypes have only been found in Brazil. The spoligotype SB1145 is the most widely-distributed, being previously reported in São Paulo [54], Minas Gerais [40], Bahia [3] and Mato Grosso do Sul [20].

The less frequent spoligotyping profiles identified in this study were SB0134, SB1136, SB1050 and SB1055, with a single isolate each. SB1136 has been described only in Brazil (Mbovis.org) [40] while SB0134 has been reported in Brazil [40,60], in Italy [43,61], in Spain [45], in France [42,62], in Algeria [46] and in the United Kingdom [58,59]. SB1050 and SB1055 were reported in the Central and Latin Americas, particularly in Argentina, Paraguay, Uruguay, Mexico, Costa Rica (Mbovis.org) and Brazil [3,40,63].

**Fig 2:**
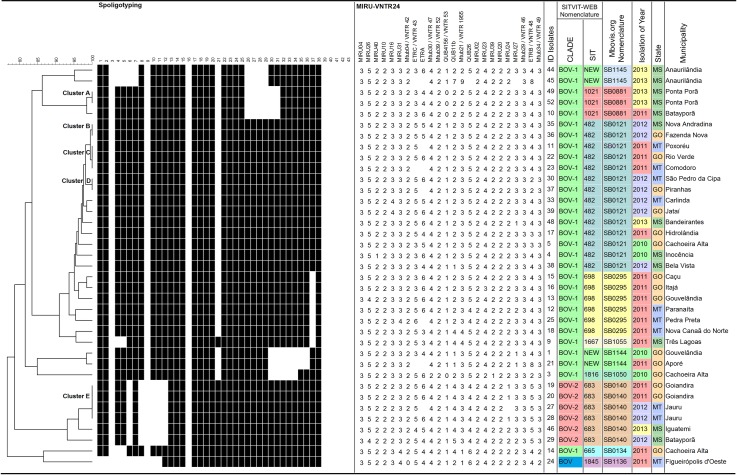
Dendrogram generated by the *BioNumerics* 6.6 software (Applied Maths) based on the combination of spoligotyping and MIRU-VNTR analyses applied to the 37 *M*. *bovis* isolates, using the categorical index and unweighted pair-grouping method analysis algorithm (UPGMA).

The 24-*loci* MIRU-VNTR patterns and the combined genotyping results are displayed in Table 2 and in Fig 2. The UPGMA based similarity of the combined genotypes are also shown.

**Table 2:** Molecular characterization of *M*. *bovis* isolates from cattle in Midwest Brazil.

Sample	Spoligotype pattern	Spoligotype Cluster	24-MIRU-VNTR profile	MIRU-VNTR cluster	Combined analyses cluster	State within Midwest Brazilian geographic region
**44**	SB1145	**Cluster S1**	352233236421225242223343	Orphan pattern	Orphan pattern	MS
**45**	SB1145	**S1**	3522332**42179*24222*38*	Orphan pattern	Orphan pattern	MS
**49**	SB0881	**Cluster S2**	352233234420225242223343	**Cluster M1**	**Cluster A**	MS
**52**	SB0881	**S2**	352233234420225242223343	**M1**	**A**	MS
**10**	SB0881	**S2**	35223323**6**420225242223343	Orphan pattern	Orphan pattern	MS
**35**	SB0121	**Cluster S3**	352233255421235242223343	**Cluster M2**	**Cluster B**	MS
**36**	SB0121	**S3**	352233255421235242223343	**M2**	**B**	GO
**11**	SB0121	**S3**	35223325*421235242223343	**Cluster M3**	**Cluster C**	MT
**22**	SB0121	**S3**	35223325**6**421235242223343	**M3**	**C**	GO
**23**	SB0121	**S3**	3522332**421235242223343	**M3**	**C**	MT
**30**	SB0121	**S3**	35223325**6**4212352422233**2**3	**Cluster M4**	**Cluster D**	MT
**37**	SB0121	**S3**	35223325*4212352422233**2**3	**M4**	**D**	GO
**33**	SB0121	**S3**	3522**4**325**5**421235242223343	Orphan pattern	Orphan pattern	MT
**39**	SB0121	**S3**	3522**4**325**6**421235242223343	Orphan pattern	Orphan pattern	GO
**48**	SB0121	**S3**	35223325**6**42123524222**1**343	Orphan pattern	Orphan pattern	MS
**17**	SB0121	**S3**	35223325**4**4212352422233**3**3	Orphan pattern	Orphan pattern	GO
**05**	SB0121	**S3**	3522332**35**4212352422233**3**3	Orphan pattern	Orphan pattern	GO
**04**	SB0121	**S3**	35**1**2332**36**421235242223343	Orphan pattern	Orphan pattern	MS
**38**	SB0121	**S3**	3522332**34**421**1**35242223343	Orphan pattern	Orphan pattern	MS
**15**	SB0295	**Cluster S4**	3522332**36**421235242223343	Orphan pattern	Orphan pattern	GO
**16**	SB0295	**S4**	3522332**36**4212352**3**2223343	Orphan pattern	Orphan pattern	GO
**13**	SB0295	**S4**	3**4**22332**5**6421235242223343	Orphan pattern	Orphan pattern	GO
**12**	SB0295	**S4**	3522332**5**642123**4**24222**2**343	Orphan pattern	Orphan pattern	MT
**25**	SB0295	**S4**	35223**4**26*42123**4**242223343	Orphan pattern	Orphan pattern	MT
**18**	SB0295	**S4**	3522332**5**3421**44**5242223343	**Cluster M5**	Orphan pattern	MT
**09**	SB1055	Orphan pattern	3522332**5**3421**44**5242223343	**M5**	Orphan pattern	MS
**01**	SB1144	**Cluster S5**	352233234421135242221343	Orphan pattern	Orphan pattern	GO
**21**	SB1144	**S5**	3522332**4211352422213**8**3	Orphan pattern	Orphan pattern	GO
**19**	SB0140	**Cluster S6**	352223256421434242213353	**Cluster M6**	**Cluster E**	GO
**20**	SB0140	**S6**	352223256421434242213353	**M6**	**E**	GO
**27**	SB0140	**S6**	35222325*4214342422*3353	**M6**	**E**	MT
**28**	SB0140	**S6**	352223256421434242213353	**M6**	**E**	MT
**46**	SB0140	**S6**	3522232**45**4214342422**2**3353	Orphan pattern	Orphan pattern	MS
**29**	SB0140	**S6**	3**4**222325**4**421**5**342422**22**353	Orphan pattern	Orphan pattern	MS
**14**	SB0134	Orphan pattern	352234254421416242222342	Orphan pattern	Orphan pattern	GO
**24**	SB1136	Orphan pattern	352234054421216242222342	Orphan pattern	Orphan pattern	MT
**03**	SB1050	Orphan pattern	352233256220235221223323	Orphan pattern	Orphan pattern	GO

*or**failed to amplify; MT—Mato Grosso state; MS—Mato Grosso do Sul state; GO—Goiás state.

While the spoligotyping resulted in six clusters containing 89.2% (33/37) of the isolates, the 24 MIRU-VNTR typing also resulted in six cluster, albeit containing only 40.5% (15/37) of the *M*. *bovis* isolates and 22 orphan patterns, demonstrating higher discriminatory power of 24 MIRU-VNTR for typing of *M*. *bovis* strains circulating in the Midwest region (Tables 2 and 3 and Fig 2).

**Table 3:** Discriminatory ability comparison among the spoligotyping and 24 MIRU-VNTR methods and the combination of both in detecting genetic similarities.

Variability	Genotyping methods		
	Spoligotyping	24*-loci* MIRU-VNTR	Combination of spoligotyping and 24-*loci* MIRU-VNTR
**Total profiles (n)**	10	28	29
**Orphan patterns (n)**	4	22	24
**Number of isolates by clusters**	2–14	2–4	2–4
**Number of grouped isolateds (n) (%)**	33 (89.2%)	15 (40.5%)	13 (35.1%)
**Discriminatory index (HGDI)**	0.810	0.980	0.982

The allele diversity of each of the 24 MIRU-VNTR *loci* is presented in Table 4. Two *loci* (ETR-A and QUB 11b) were the most discriminatory (h = ≥ 0.50), while six presented moderate allelic diversity (MIRU 16, MIRU 27, ETR-B, ETR-C, Mtub21, QUB 26; h index between 0.33 to 0.49). Low allele diversity (h = ≤ 0.15) was observed for eight MIRUs and no diversity at all in another eight markers (Table 4). This means that eight MIRUs should be sufficient for the genotyping study of the *M*. *bovis* isolates from the Brazilian Midwest.

**Table 4:** Allele diversity of the 24-*loci* MIRU-VNTR.

	Number of repetitions										
*Locus*	0	1	2	3	4	5	6	7	8	9	Allele diversity (HGDI) (h index)
**MIRU 02**			37								0.00
**MIRU 04**				37							0.00
**MIRU 10**			37								0.00
**MIRU 16**			6	29	2						**0.36**
**MIRU 20**			37								0.00
**MIRU 23**			1	1	35						0.10
**MIRU 24**		3	33								0.15
**MIRU 26**					2	35					0.10
**MIRU 27**		3	4	29							**0.33**
**MIRU 31**				34	3						0.15
**MIRU 39**		1	36								0.05
**MIRU 40**		1	36								0.05
**ETR-A**				2	8	5	15				**0.65**
**ETR-B**			3	2	25	6			1		**0.48**
**ETR-C**				10	1	22	1				**0.49**
**Mtub 04**	1		36								0.00
**Mtub 21**		2	4	28	2					1	**0.38**
**Mtub 29**				37							0.00
**Mtub 30**			1		36						0.05
**Mtub 34**			2	34							0.11
**Mtub 39**			37								0.00
**QUB 11b**		3	24		8	1		1			**0.52**
**QUB 26**					8	26	2				**0.43**
**QUB 4156**	4	33									0.00

These results corroborate with earlier data, which showed high resolution of ETR-A, ETR-B and ETR-C in the genotyping of *M*. *bovis* isolates from the state of Rio de Janeiro [64]. High resolution of ETR-A and ETR-B was also observed in Chad [23], Belgium [26] and Italy [43], proving their ample discriminatory power for *M*. *bovis* isolates, epidemiologically related or not [26,43].

Previous studies described the resolving power of the aforementioned ETRs (ETR A-F) [65] and QUBs (Queen's University Belfast VNTRs) [53], which are part of the 24-*loci* MIRU-VNTR set. Campos et al. (2013) [66] found a similar discrimination of *M*. *bovis* strains in Spain by evaluating the QUB 26 *locus*, but lower allelic discrimination by QUB 11b. Both *loci* were highly discriminative in Belgium [26], but only moderately discriminative in Italy [43].

Both MIRU 16 and MIRU 26 *loci* were highly discriminative in the study by Parreiras et al. (2012) [40], different from the present study. The MIRU 16 *locus* was considered inefficient for the differentiation of *M*. *bovis* strains in Ireland [67], Italy [43] and Portugal [27]. While Hilty et al. (2005) [23] and Allix et al. (2006) [26] described the MIRU 27 *locus* as highly discriminatory for *M*. *bovis* strains isolated in Chad and Belgium, Boniotti et al. (2009) [43] claimed this *locus* to be ineffective to characterize *M*. *bovis* isolates from Italy.

The lack of discriminatory power of MIRU 02, MIRU 10, MIRU 20, MIRU 23, MIRU 24, MIRU 31 and MIRU 39 was also demonstrated by Figueiredo et al. (2011) [64] and Parreiras et al. (2012) [40], both in Brazil, and by Roring et al. (2004) [67], in Ireland. Lack of differentiation by MIRU 02, MIRU 04, MIRU 10, MIRU 20, MIRU 23, MIRU 31, MIRU 39 and MIRU 40 *loci* was observed in Belgium [26] and Italy [43].

Although some individual *loci* show great discriminatory power, both for *M*. *tuberculosis* and *M*. *bovis* isolates, in general the *loci* are less polymorphic in *M*. *bovis*. Thus, it is better to combine distinct and use individual combinations of genotyping markers in each geographic study area [23,67].

Previous studies conductes in the South and southeastern regions of Brazil analyzed the genetic variability of *M*. *bovis* isolates from 12 to 15-*loci* from MIRU-VNTR [19,40,64]. In the present study, the allelic diversity and, consequently, the discriminatory power of the 24 MIRU-VNTR *loci* in a convenience sample obtained in the Midwest Braizlian region, from 2010 to 2013, were investigated for the first time.

Spoligotyping showed a discriminatory index of 0.810 (Table 3), similar to previous studies [26,40,45], but higher than that described by Ramos et al. (2014) [19]. The 24-MIRU-VNTR typing, on the other hand, provided a discriminatory index of 0.980 and the combination of the methods presented a discrimination of 0.982 (Table 3), higher than those observed by Sola et al. (2003) [25], Parreiras et al. (2012) [40] and Ramos et al. (2014) [19]. Roring et al. (2002) [22] and Hilty et al. (2005) [23], when evaluating *M*. *bovis* isolates from Europe and Africa, showed that the MIRU-VNTR technique has greater ability to discriminate *M*. *bovis* isolates compared to spoligotyping. The slight difference in efficiency observed with or without adding spoligotyping to 24-MIRU-VNTR typing demonstrates that this technique by itself would be able to differentiate between *M*. *bovis* strains in the Brazilian Midwest. The main limitation of spoligotyping is that all genetic polymorphisms are restricted to a single genomic *locus*, the DR region, which limits resolution. While having the advantages of being considerably faster, spoligotyping alone still does not provide sufficient discrimination between *M*. *bovis* strains to be used as a sole typing method, and it is, thus, often combined with supplementary techniques [56,57,67].

MIRU-VNTR is considered the gold standard for MTC genotyping, since it is highly disriminatory and reproducible [68]. Its repeating units are located in *loci* scattered throughout the genome of MTC strains [31], with variable mutation rates for each *locus* [32,69]. The polymorphism of the strains is based on the variability of the number of copies of each repeating unit. The original MIRU-VNTR methodology included 12-*loci* was used in conjunction with spoligotyping for the first MTC genotyping. However, its discriminatory power was less than IS*6110* RFLP [69,70]. Due to the low discriminatory power of the MIRU-VNTR 12-*loci*, current studies suggest the use of a set of 15-*loci* for molecular epidemiological studies and 24-*loci* for phylogenetic studies [32]. Currently, the method has a high yield due to multiplex-PCR application using primers labeled with different fluorophores. This amplified material is subjected to capillary electrophoresis in an automatic sequencer, to estimate the size of the PCR product [71,72]. The advantage of automated typing by MIRU-VNTR is the fact that method is highly reproducible, faster and less laborious than the original methodology, yielding more reliable results because of the computerized analysis of the generated fluorescent signals.

There is a consensus among different studies that, by associating the results of spoligotyping to those obtained by MIRU-VNTR, discrimination between strains is more effective, and, thus, the combination of methodology has been considered the best strategy for the molecular typing of *M*. *bovis* [21]. In addition, Sola et al. (2003) [25], Allix et al. (2006) [26] and Duarte et al. (2010) [27] demonstrated that the combination of these techniques has a good cost/benefit ratio due to speed, reproducibility and reliability of *M*. *bovis* genotyping.

Better discrimination between *M*. *bovis* strains by combining the spoligotyping and MIRU-VNTR results has also been described by Ramos et al. (2014) [19]. Figueiredo et al. (2011) [64] indicated considerable genetic variability between 12 isolates of *M*. *bovis* originated from a herd of 34 tuberculin-positive cows in the state of Rio de Janeiro. The authors grouped the isolates in two clusters and six orphan patterns. In another study [40], where 61 isolates from the five Brazilian macro regions (South, Southeast, Midwest, North and Northeast) were analyzed by spoligotyping and 12-*loci* MIRU-VNTR, the isolates were grouped in eight clusters containing 53 isolates and eight orphan patterns, confirming the genetic variability of *M*. *bovis* strains in the country.

Herein, five clusters with 13 isolates (35%) (Fig 2) were observed and interestingly, strains with orphan patterns were found predominantly in the state of Goiás (10/24), besides the clustered strains. In the state of Mato Grosso, clusters "C", "D" and "E" were found, along with 5 orphan patterns. Finally, in the state of Mato Grosso do Sul, strains were clustered in "A" and "B" and nine of them showed orphan patterns. Epidemiologically related isolates are derived from the clonal expansion of a single precursor and as a result, have common characteristics that differ from those that are unrelated epidemiologically [64].

The great genetic heterogeneity of *M*. *bovis* observed in the Brazilian Midwest can be explained by the animal movement that occurs between different regions and farms, thus causing the spread of numerous *M*. *bovis* strains in the herds of the region. Another important point to consider is that the Midwest region of the country is a dry border with other Latin American countries, such as Bolivia and Paraguay, over a wide range of territory, thus allowing contact between herds of both countries, resulting in the transfer of *M*. *bovis* strains to Brazil, which can be retained in the Midwest region, or possibly migrate to other, more remote, regions.

In the present study, the association of spoligotyping and 24-MIRU-VNTR for the molecular characterization of *M*. *bovis* isolates from the Brazilian Midwest was carried out for the first time and indicated that BTB in this geographical region is caused by *M*. *bovis* isolates with high genetic diversity, which may hinder *in vivo* diagnosis, control and eradication of the disease. The characterization of *M*. *bovis* circulating genotypes in the geographical region aids in tracking and sanitizing remaining outbreaks of disease, since BTB has a low prevalence in this region of Brazil.

## Conclusions

Ten spoligotypes are present in the Brazilian Midwest region. The combination of spoligotyping with the 24-MIRU analysis rendered five clusters and 24 orphan patterns, confirming the high genotypic diversity among *M*. *bovis* strains circulating in the Midwest Brazil. The presence of different *M*. *bovis* genotypes in this region suggests movement of animals between regions or different sources of infection. Thus, it is possible to conclude that BTB in the Brazilian Midwest is caused by multiple *M*. *bovis* strains.
